# Supplementary material for: Complexome profiling of the Chlamydomonas psb28 mutant reveals TEF5 as an early PSII assembly factor
Source: Plant Cell. 2025 Mar 17;37(6):koaf055. doi: 10.1093/plcell/koaf055 (PMC12164590; doi:10.1093/plcell/koaf055)
Supplement: koaf055_Supplementary_Data [file koaf055_supplementary_data.zip › psb28_interactive.zip!/plots/intensity/qualityPlots/peptides/NUO6_Cre10.g422600.t1.1.html]

### Cre10.g422600.t1.1

Trivial name: NUO6  
SeqCoverage [median]: 25.00  
Mass: 52.23 kDa  
  
 

| Sequence | MissedCleavage | Mass [Da] | wtIntensity | muIntensity | Score |
| --- | --- | --- | --- | --- | --- |
| AGYIYIR | 0 | 854.47 | 21841.71 | 26370.45 | 130.84 |
| AISEAYAK | 0 | 851.44 | 17455.33 | 29411.11 | 153.73 |
| EAQSGLGTAAVIVMDK | 0 | 1588.81 | 6549.38 | 8077.08 | 186.94 |
| GEFVQER | 0 | 863.41 | 22018.98 | 17370.29 | 111.73 |
| GGAGFPSGLK | 0 | 889.47 | 0.00 | 2593.98 | 68.22 |
| GPEWFSSFGR | 0 | 1168.53 | 2081.13 | 0.00 | 101.64 |
| HFRGEMEER | 1 | 1189.53 | 0.00 | 5273.58 | 56.01 |
| IFQNIYGR | 0 | 1009.53 | 15763.33 | 10895.26 | 162.07 |
| LEEIDMLWEITK | 0 | 1518.76 | 486.12 | 0.00 | 68.79 |
| RGPEWFSSFGR | 1 | 1324.63 | 6234.28 | 6745.50 | 72.34 |
| STDVIDAIAR | 0 | 1059.56 | 66960.06 | 64540.98 | 223.88 |
| TSFGGLKDEDR | 1 | 1223.58 | 11534.96 | 7620.56 | 79.66 |
| TSFGGLKDEDRIFQNIYGR | 2 | 2215.10 | 5217.43 | 7553.25 | 91.46 |
|  |  |  | 176142.7 | 186452.0 |  |
